# Cartesian three-dimensional method to quantify displacements between cone beam computed tomography models

**DOI:** 10.1590/2177-6709.27.5.e222199.oar

**Published:** 2023-01-06

**Authors:** Caroline Pelagio Maués CASAGRANDE, Marcus Vinicius Sena CASAGRANDE, Andressa Otranto de Brito TEIXEIRA, David Silveira ALENCAR, Bruno Santos de Barros DIAS, Rhita Cristina Cunha ALMEIDA, Cátia Abdo QUINTÃO, Felipe Assis Ribeiro CARVALHO

**Affiliations:** 1 Universidade do Estado do Rio de Janeiro, Faculdade de Odontologia (Rio de Janeiro/RJ, Brazil).; 2 Universidade Federal do Rio de Janeiro, Faculdade de Odontologia (Rio de Janeiro/RJ, Brazil).; 3 Hospital de Reabilitação de Anomalias Craniofaciais, Universidade de São Paulo (São Paulo/SP, Brazil).

**Keywords:** Orthognathic surgery, Orthodontics, Imaging, three-dimensional

## Abstract

**Introduction:**

Research in Orthodontics and Oral Surgery has been relying on three-dimensional (3D) models to evaluate treatment results with displacement color map techniques, even though it has important limitations.

**Objectives:**

This study proposed a method of tracking translational movements of 3D objects to evaluate displacements in surfaces with no shape modification.

**Methods:**

Cone Beam Computed Tomography (CBCT) data of ten patients were imported to the Dolphin software. A hypothetical virtual surgical plan (randomly defined) was developed in the software and afterwards verified using the proposed method. All the procedures were carried out by two evaluators, in two different time-points, with a 15-day interval. ITK-Snap software was used to generate high quality STL models. Centroid points were automatically generated and their coordinates were compared to confirm if they represented the known displacements simulated. The paired *t*-test and the Bland-Altman plots were used, as well as the intraclass correlation coefficient.

**Results:**

Interexaminers and intra-examiner tests showed excellent reliability of the method, with mean displacement measurement error values under 0.1mm. The paired *t*-test did not show any statistically significant differences.

**Conclusion:**

The method showed excellent reliability to track the simulated translational displacements of bone segments.

## RESUMO

**Introdução:**

Grande parte das pesquisas em Ortodontia e Cirurgia Oral tem utilizado modelos tridimensionais e realizado avaliações por meio de mapa de cores de deslocamento, apesar das limitações existentes.

**Objetivo:**

O presente estudo teve como objetivo propor um método de rastreamento de movimentos translacionais de objetos 3D, para avaliar deslocamentos em superfícies sem modificação de forma.

**Métodos:**

Dez adultos que seriam submetidos à cirurgia ortognática tiveram suas tomografias computadorizadas de feixe cônico pré-cirúrgicas importadas para o *software* Dolphin. Um plano cirúrgico virtual hipotético com deslocamentos conhecidos foi desenvolvido e posteriormente verificado pelo método proposto. Todos os procedimentos foram realizados por dois avaliadores, em dois momentos diferentes, com intervalo de 15 dias. O *software* ITK-Snap foi usado para gerar modelos STL de alta qualidade dos ossos do paciente. Os pontos do centroide foram gerados automaticamente, e suas coordenadas foram comparadas, para confirmar se representavam os deslocamentos conhecidos simulados. Para análise estatística, foram usados teste *t* pareado, coeficiente de correlação intraclasse e os gráficos de Bland-Altman.

**Resultados:**

O teste inter e intraexaminadores mostrou boa confiabilidade do método, com valores médios abaixo de 0,1mm para os erros de medida de deslocamento.

**Conclusão:**

O método mostrou boa confiabilidade para avaliar o deslocamento de segmentos ósseos após cirurgia ortognática, devendo ser aplicado como uma ferramenta completa de avaliação verdadeiramente tridimensional.

## INTRODUCTION

The gold standard for the orientation and comparison of different tomographic time points is the voxel-based computed tomography superposition, introduced in Dentistry by Cevidanes et al.^1^ This method uses the existing grayscale differences in voxels to perform the alignment of two images, without the need to rely on operator landmark placement.^2^

Before the assessment is performed, it is necessary to segment the tomographic model to be studied, generating a reliable three-dimensional (3D) model in Standard Triangle Language (stl). For this procedure, semi-automatic segmentation is the gold standard. When it comes to the quantification of differences among these superimposed models, the commonly used methods are: anatomical landmarks comparisons, color maps and shape correspondence. Furthermore, these methods present deficiencies that may influence the reliability and/or practicality of the analysis.^3^

The first 3D image evaluations used anatomical landmarks comparisons, as often used to track changes in Orthodontics when analyzing two-dimensional images. Linear and angular measurements to describe surface changes have also been widely used since the pioneer studies using 3D tomographic models. However, landmark placement is critical for both methods, requiring calibrated evaluators to produce accurate measurements.^3^

Color maps became a popular method of comparing 3D models. This quantification method plots, graphically, the distances between homologous points on the surfaces of models to be compared. Although this technique intuitively illustrates regional changes, and software tools allow the user to measure the mean point distances of a given area, its major drawback is that homologous points are usually determined by Iterative Closest Point (ICP) algorithm, which uses the smallest point-to-point distance between two surface models, and frequently results in an incorrect correspondence between anatomical structures. This issue is even more evident when the evaluated anatomical structures present marked curvature areas, as the mandible. Furthermore, it is not possible to assess the direction of changes.^4^

Trying to overcome this issue, the shape correspondence method was developed to evaluate virtual 3D surface models, mapping similar structures and identifying correspondent anatomical points using the first order ellipsoid from the spherical harmonic coefficients. The spherical parametrizations are aligned to establish correspondence across all surfaces. This method is reliable and overcomes the limitations of the ICP, although it lacks a proper graphical user interface (GUI) and is extremely time-consuming, which constrains its routine application.^5^

Thus, due to the limitations of the existing methods, the present study aimed at describing and validating a novel and efficient methodology for tracking translational (vertical, sagittal and transversal) displacements between 3D models of anatomical regions. 

## MATERIAL AND METHODS

This project was approved by the Research Ethics Committee of the Pedro Ernesto University Hospital, under the protocol number CAAE: 46729315.3.0000.5259. The informed consent was obtained from all participants. A sample size calculation was performed considering a power of 95% and a level of significance of 0.05, to detect a difference of 0.4 mm of the centroid translations, on any of the axes (X, Y and Z). As result, nine patients would be necessary to perform the analysis. The used value of 0.4mm represents commonly used voxel size (full-head CBCT scans), so that differences smaller than this are considered intrinsic to the CBCT evaluation.^6^

The sample consisted of 10 adult patients with full-head CBCT acquired with Classic i-CAT scanner (Image Sciences, Hatfield, PA, USA) extracted from files of a maxillofacial surgery clinic (presurgical scans). The mean parameters used to acquire the images were: voxel dimension of 0.4 mm (isometric), with 40 seconds exposure, tube voltage of 120 (±5) kV and tube current of 3-8 (± 10%) mA.

This method was designed to quantify the displacement between two tomographic regions. It is worth remembering that there can be no change in the shape of the bone structure, that is, the patient cannot be growing. Thus, the indication of this method would be patients who underwent orthognathic surgery, or to evaluate the skeletal result of some orthodontic mechanics, for example. The first step is the voxel-based superposition at the cranial bases, followed by the selection of the region of interest in each tomography (maxilla and mandible), and calculating the centroid of this structure. The displacement will be quantified by subtracting the coordinates of each centroid.

**Figure 1:**
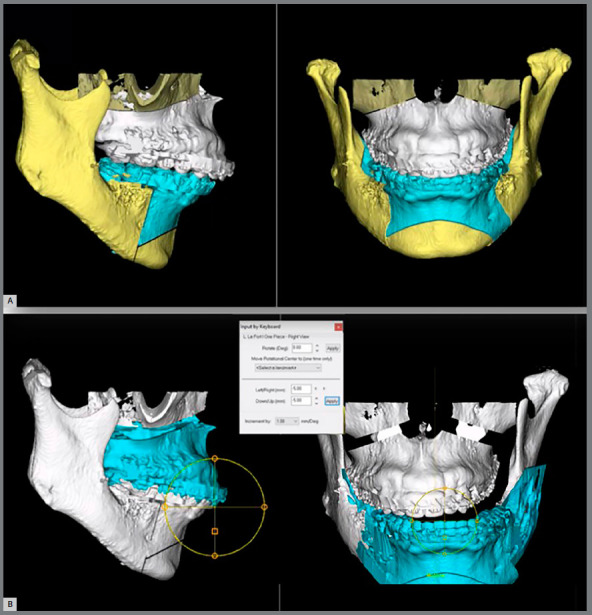
Example of a creation of a HVSP, using the displacement values randomly chosen with no concern in obtaining an ideal occlusal result: A) Initial and B) post-HVSP.

To check the method’s reliability for identifying the translational displacements of skeletal segments in orthognathic surgical patients, an analysis was performed based on the following steps:

1. Head orientation - Each CBCT was imported to Dolphin Imaging software (Dolphin Imaging and Management Systems, Chatwirth, CA, USA) and head orientation was performed according to the patient’s natural head position (NHP).^7^ Frontal and profile photographs, with the patient in their natural positioning (looking inside their eyes on a flat mirror), were used to help the operator to orient the tomographies in the software. This step is crucial to standardize the reference plans orientation for surgical movements performed in the next step.^8^

2. Creation of a Hypothetical Virtual Surgical Planning (HVSP) - When comparing pre- and post-surgical tomographies, for example, it is not possible to assess whether the changes that occurred are inherent to the surgical procedure or whether the differences are due to other factors such as the surgeon’s expertise. Thus, to validate the methodology, it was decided to simulate a surgical plan, thus removing any possible confounding factor. A VSP was carried out with all patients, simulating a hypothetical bimaxillary surgery by means of a 1-piece maxillary LeFort I and a bilateral sagittal split osteotomy (BSSO) for the mandible. Displacement values for each patient’s maxillary and mandibular segments HVSP were randomly chosen (Table 1) for the three planes of space (sagittal - X-axis; vertical - Y-axis; transversal - Z-axis) using the iOS random 1.1 application (Mireia Lluch Ortola). There was no concern in obtaining an ideal occlusal result (Fig 1), while all HVSP aimed to represent as far as possible the range of surgical movements, respecting the limits described by Proffit’s discrepancy envelope. At the end of this process, .stl files of the maxillary and mandibular segments (initial and post-HVSP) of each patient were exported. This step was performed in Dolphin imaging software and aimed to test if the proposed method could be used to verify the simulated surgery values. 

**Table 1:** Maxillary and mandibular displacement values randomly chosen.

	Simulated movement						Error					
	Maxilla			Mandible			Maxilla			Mandible		
	X	Y	Z	X	Y	Z	X	Y	Z	X	Y	Z
Patient 1	-2.5	7.5	-2	4.4	7.5	5	0.06	0.06	-0.23	0.05	-0.16	-0.16
Patient 2	5	5	4	20.8	10	-5	-0.01	-0.08	-0.08	-0.07	0.16	-0.14
Patient 3	7.5	-7.5	-5	-7.6	-15	3	-0.12	0.02	0.21	0.08	-0.01	-0.16
Patient 4	10	-5	-4	12.6	-5	2	0.00	0.03	0.08	0.01	0.00	0.08
Patient 5	15	-10	-1	-12	2.5	1	0.66	0.89	-0.08	-0.18	-0.73	0.08
Patient 6	2.5	-15	5	0.3	-10	-3	-0.01	0.04	-0.10	0.01	-0.05	-0.04
Patient 7	-10	10	2	-3.8	-7.5	-2	-0.02	-0.01	0.06	0.00	-0.02	0.16
Patient 8	-7.5	2.5	1	8.5	-12.5	-4	-0.02	-0.04	0.04	0.09	0.05	-0.05
Patient 9	5	-12.5	3	25	-2.5	4	-0.03	0.00	-0.10	-0.03	-0.01	-0.16
Patient 10	12.5	-2.5	-3	16.7	5	-1	0.03	-0.42	-0.06	-0.03	0.10	-0.06

**Figure 2:**
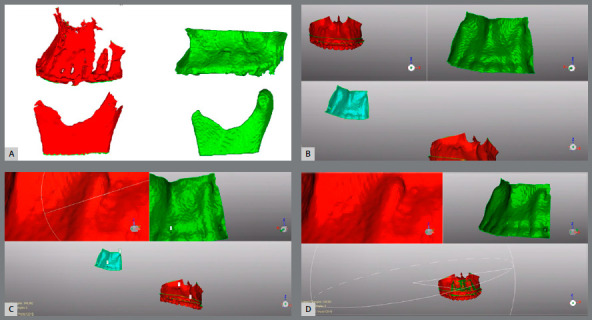
In **A**, it is possible to observe the differences between the models generated by Dolphin (red) and ITK (green) softwares. Images **B**, **C**, and **D** shows the sequence for best fit alignment in Geomagic software.

3. Creation of an accurate 3D model - 3D models produced by Dolphin^®^ are good enough for visualization purposes and to determine the bone fragments spatial position, but are not accurate enough to represent the exact shape of a bone segment, since the 3D model generation is based simply on limited voxel intensity thresholding (Fig 2A). The creation of the best possible 3D models can be achieved by the semi-automatic CBCT segmentation.^9^ Therefore, it was used the semi-automatic segmentation procedures in ITK-SNAP software (Cognitica, Philadelphia, Pa), which utilizes active contour methods to compute anatomic structures based on the CBCT image gray level intensity and boundaries. In this way, accurate surface models of the regions of interest were also exported as .stl files. 

Steps 4 to 7 comprise the actual methodology proposed and tested on this study, and will be necessary whenever it is used to track 3D objects translations.

**Figure 3:**
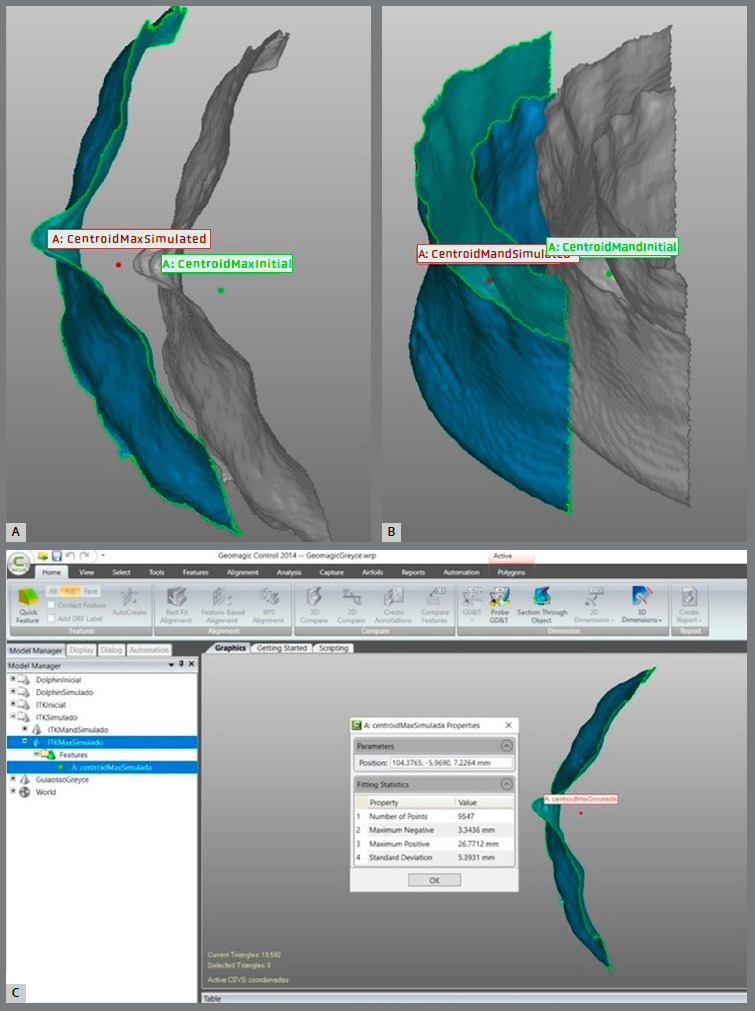
Visualization of the initial and simulated centroid of the maxilla (**A**) and the mandible (**B**), and analysis of the X, Y, and Z positioning of the centroid in the simulated maxilla (**C**).

4. Aligning models - Geomagic Qualify 2013 (3D Systems, Rock Hill, SC) was used to align (best fit) the reliable surface models generated by the ITK-SNAP software (v. 3.6; Cognitica, Philadelphia, Pato) to the less precise Dolphin models (used as a spatial reference). From this point on, the ITK models oriented according to their Dolphin counterparts were used as the models to be evaluated (Fig 2). To assess this superimposition, the RMS (root mean square) was analyzed.

5. Creation of a Cartesian Coordinate System - In this step, it was important to use the same Cartesian coordinate system (CCS) used in Dolphin software (based on sagittal, coronal and axial planes) in Geomagic Qualify software. Therefore, a CCS was created in Dolphin and exported.

6. Creation of the centroid point - To quantify anatomical regions of interest (ROI) translational displacements in a systematic way, the 3D centroid of each surface was automatically created by the software, to represent each ROI spatial position (Fig 3). To safely use the centroid as a reference for a spatial position, it is mandatory to crop the ROI time points with the same boundaries. Also, the meshes need to be even and with a similar number of triangles.

7. Displacement quantification. Finally, the centroid’s spatial position was registered (X, Y, and Z axis), according to the CCS, for initial and HVSP maxillary and mandibular models, allowing the calculation of translational displacements (Fig 3C). This step was conducted in Geomagic Qualify 2013 software (3D Systems,Rock Hill, SC). 

**Figure 4:**
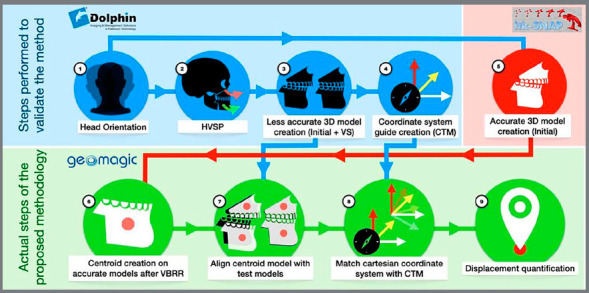
Flowchart of the procedures performed.

All procedures were carried out by two evaluators, in two different time points, with a 15-day interval, with the exception of the head positioning, which was performed by the maxillary surgeon responsible for the patient. The flow chart presented in Figure 4 illustrates the procedures step by step.

## STATISTICAL ANALYSIS

A statistical analysis was performed with the use of the Statistical Package for the Social Sciences (SPSS^®^, version 23.0) software application for Windows (Armonk, NY: IBM Corp). The normal distribution of samples was assessed by the Shapiro-Wilk test. The intraclass correlation and Bland-Altman plots were used to determine intra and inter-examiners reproducibility. The paired*t*-test was used to compare the known displacements created by the HVSP, and the translations measured by the proposed method. The Bland-Altman plots were also used to compare the mean differences between both methods. 

## RESULTS

Mean differences between the known HVSP and the translational displacements measured by the proposed method showed discrepancies smaller than 0.1mm for all evaluated situations (for both examiners) (Tables 2 and 3). The paired *t*-test did not show any statistically significant differences (Tables 2 and 3). Intraclass correlation coefficients revealed excellent intra- and inter-examiner reproducibility, and the mean difference and confidence interval showed values smaller than 1 mm (Table 3). 

**Table 2:** Descriptive statistics and paired *t*-test comparing the differences between the VSP performed in Dolphin and the method using centroid, found by Evaluator 1, considering the displacement direction.

		Paired differences (Evaluator 1)						
		Mean	Standard deviation	Mean error	95% Confidence interval of the difference		T	P
					Lower	Upper		
Maxilla	Sagittal (X)	-0.06	0.219	0.07	-0.21	0.10	-0.79	0.45
	Vertical (Y)	-0.05	0.33	0.10	-0.28	0.19	-0.46	0.66
	Transversal (Z)	0.03	0.12	0.04	-0.06	0.12	0.67	0.52
Mandible	Sagittal (X)	0.01	0.08	0.03	-0.05	0.06	0.28	0.79
	Vertical (Y)	0.07	0.25	0.08	-0.11	0.25	0.84	0.43
	Transversal (Z)	0.05	0.12	0.04	-0.04	0.13	1.20	0.26

Values measured in millimeters.

**Table 3:** Descriptive statistics and paired *t*-test comparing the differences between the VSP performed in Dolphin and the method using centroid, found by Evaluator 2, considering the displacement direction.

		Paired differences (Evaluator 2)						
		Mean	Standard deviation	Mean error	95% Confidence interval of the difference		T	P
					Lower	Upper		
Maxilla	Sagittal (X)	-0.09	0.20	0.06	-0.23	0.05	-1.39	0.20
	Vertical (Y)	-0.08	0.26	0.08	-0.27	0.11	-0.93	0.38
	Transversal (Z)	0.03	0.09	0.03	-0.03	0.09	1.05	0.32
Mandible	Sagittal (X)	0.01	0.07	0.02	-0.04	0.06	0.52	0.61
	Vertical (Y)	0.07	0.22	0.07	-0.09	0.23	1.01	0.34
	Transversal (Z)	-0.03	0.10	0.03	-0.10	0.05	-0.79	0.45

Values measured in millimeters.

**Table 4:** Intraclass correlation coefficient and Bland-Altman limits of agreements.

	Intraclass correlation coefficient						Mean difference					
	Maxilla			Mandible			Maxilla			Mandible		
	X	Y	Z	X	Y	Z	X	Y	Z	X	Y	Z
Interexaminer	1.000	1.000	1.000	1.000	0.999	1.000	-0.309	-0.306	0.003	0.004	0.003	-0.069
Intraexaminer 1	1.000	0.999	1.000	0.999	0.999	1.000	0.047	0.047	0.042	-0.015	-0.057	-0.009
Intraexaminer 2	1.000	1.000	1.000	1.000	0.999	1.000	0.024	-0.015	-0.057	-0.003	-0.00162	0.031
	95% Confidence Interval (Upper)						95% Confidence Interval (Lower)					
	Maxilla			Mandible			Maxilla			Mandible		
	X	Y	Z	X	Y	Z	X	Y	Z	X	Y	Z
Interexaminer	0.007	0.070	0.113	0.0449	0.064	0.0480	-0.069	-0.132	-0.107	-0.035	-0.056	-0.187
Intraexaminer 1	0.0225	0.322	0.158	0.070	0.151	0.1209	-0.131	-0.227	-0.072	-0.100	-0.266	-0.139
Intraexaminer 2	0.083	0.027	0.056	0.36	0.79	0.149	-0.035	-0.058	0.1709	-0.044	-0.083	-0.0871

The Bland-Altman graphics were used to illustrate the differences between the methods recorded by evaluator 1. 

**Figure 5:**
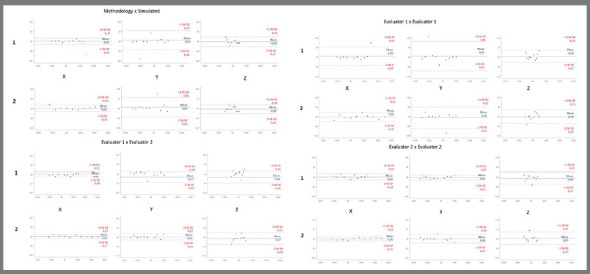
Image illustrating the Bland-Altman graphs, showing the results concerning sagittal (X), vertical (Y) and transversal (Z) movements for the maxilla and mandible (1 and 2, respectively) and the intra and inter-examiners reproducibility.

In the maxilla, it was observed that for sagittal and vertical movements the mean difference was -0.05 mm (Fig 5, 1X, 1Y). For those two directions, nine out of ten values were very close to the average, and one was an outlier. For transversal movements (Fig 5, 1Z) the mean difference was 0.03 mm. For the mandible, the mean differences were: 0.01 mm for sagittal movements (Fig 5, 2X); 0.06 mm for vertical movements (Fig 5, 2Y) and 0.04 mm for transversal movements (Fig 5, 2Z). 

Even the outliers values were smaller than 1 mm, which are very close to the spatial resolutions of CBCT used in the present study (Fig 5, 1 and 2).

In Bland-Altman plots, used to determine intra and inter-examiners reproducibility, it can be seen that the 95% limit of agreement does not exceed 0.7mm, showing an excellent result. The “Z” axis showed a greater difference, in comparison with the “X” and “Y” axes, between examiners 1 and 2. In another hand, the “Y” axis showed a greater difference between two-time points for examiner 1.

To check the quality of superimposition, the RMS (root mean square) was used to assess the differences in positioning between the models (Dolphin and ITK). The RMS corresponds to the absolute average of the distances in a normalized way. The RMS found is 0.22 ± 0.12 (mean and standard deviation of RMS, in mm), showing that both models were properly superimposed.

## DISCUSSION

Automatic or semiautomatic CBCT segmentation is key in order to build reliable 3D models.^2^ ^-^ ^4^ Quantitative evaluations should be translational or rotational, based on the different axes (X, Y, and Z).^2^

In this research, a surgical simulation was performed with the Dolphin Imaging^®^ VSP module, due to its marketing popularity and availability, as well as the author’s proficiency with this software. An important drawback of the Dolphin, however, is that although the created 3D model precisely represents the anatomical spatial position, it does not accurately represent its shape. To overcome this limitation, all the quantifications carried out in this study were based on semiautomatic segmentation models created with ITK-SNAP^®^ software (v. 3.6; Cognitica, Philadelphia, Pa).

Using two models from different origins, in the present study, represented that it was necessary to align the .stl files from different sources, despite the fact that the same CBCT scans were used for segmentation, in both software. As the quality of those files generated with Dolphin was lower, this could add imprecision to their alignment with the ITK’s models, which could contribute to an increase in the differences between the simulated and the measured values. However, the results obtained showed that these changes were not relevant, and the method proved to be reliable.

Bone displacements visualization aided by Iterative Closest Point (ICP) algorithm is advisable for qualitative evaluation of two different time points. However, its main disadvantage is not showing the differences between the corresponding points. This limitation is more significant when large displacements, rotations and/or bone remodeling occur. Furthermore, this method does not indicate vector changes in the corresponding anatomical regions.^8^

The shape correspondence method is now the gold standard in the evaluation of morphological changes due to pathological processes, growth changes, and skeletal displacements. The method described in this study is limited when compared to the shape correspondence, due to the impossibility to verify displacements in structures that would undergo shape modifications (for instance, when evaluating growing patients). On the other hand, although shape correspondence is freely available through the SPHARM-PDM toolbox, it lacks a proper graphical user interface (GUI), and its workflow is extremely time-consuming, therefore limiting its broader application.^5^ ^,^ ^10^

Considering the application of this method in a real-world environment of a pre- *versus* post-surgical sample, it could be suitable for nongrowing individuals if the anatomical regions of interest did not suffer any morphological changes; for instance, in a mandibular advancement surgery, the overall shape of the mandible is altered by the procedure, but the subregions like the chin or the condyles keep their shape in the short term. Caution should be taken in order to use the current method for longer follow-ups. There is a lack of evidence that the ROIs are morphologically stable in the long term.

Some studies tried to access translational changes decomposing displacements on different axes of space,^11^ ^,^ ^12^ but the methodologies used were dependent on the operator for landmark placement, which incorporates errors in the measurements. 

In addition, the studies that evaluated the clinical results produced by existing VSP software, analyzed tomographic images of patients who underwent orthognathic surgery.^10^ ^,^ ^12^ Their results could be considered biased, since the surgical technique used, the surgeon training level, and the patient’s individual response would act as confounding factors against the VSP accuracy evaluation. The present study attempted to eliminate this bias, which could interfere with the results of simulated surgeries.

In Bland-Altman plots, between examiner 1 and 2, although the difference in Z axis was greater than on the X and Y axis, this difference remained small, with an average of -0.18mm. In another hand, the “Y” axis showed a greater difference between two-time points in examiner 1. This was due to the measurements of a patient that had a greater variation. Despite this, the differences remained small.

Another critical issue is the definition of NHP,^8^ since inconsistencies in this orientation between two time points can lead to inconsistent measures, especially if the image evaluation involves the decomposition of the translations on the X, Y, and Z axes, or if the two-dimensional images are reconstructed from 3D data sets. Ideally, the head position should be saved, so that each image of each subject follows the same reference planes, allowing these images to be compared.^8^ In this study, the definition of NHP was performed using photographs taken from the patient with a focus on the distant horizon,^7^ with subsequent positioning of the CBCT according to the photographs in the Dolphin. Although there are other methods for this determination, such as the use of facial landmarks,^13^ systems of algorithms to calculate the rotations of objects, or laser scanners,^14^ all of these methods present some validation problem, in addition to variations in cost and practicality.^13^

The use of the 3D centroid in this study is advantageous when compared to manual landmark placement, since it is automatically and systematically generated by the software. The centroids of each anatomical region for each time point could be described by the X, Y, and Z coordinates, and displacements could be calculated by the subtraction of the coordinate values between time points. Most of the studies that evaluated VSP in the literature used manual landmark placement, which is not ideal.^11^ ^,^ ^15^ ^-^ ^19^ Even in a case when a centroid was used, it was based on a triangle determined by the vertices, which were manually defined.^17^ A limitation of the centroid point is that it does not allow evaluation of rotational movements.

In this study, the mean error was less than 0.1 mm. Even the extreme errors, represented by the outliers, were smaller than 1 mm, which is very close to the spatial resolution of the CBCT (0.7 mm),^6^ and below the clinically relevant limit, commonly considered as 2.0 mm.^11^ Thus, this method can identify the translations undergone by 3D structures without adding relevant error to the sensitivity of the CBCT scan, with excellent inter-examiner reliability. 

The proposed method could enable the 3D evaluation of anatomical structures in several studies, such as the results of orthognathic surgery,^20^ illustrating the difference between what was planned and what was achieved with osteotomies; although it needs to be validated for long-term follow-up evaluations, when bone remodeling might influence centroid translations. Assessing changes in tooth positions after orthodontic treatments is also viable, making it possible to identify limitations and side effects of the mechanics used. It might be important to notice that this method is applicable to any stl file, what would make it possible to assess tooth position changes with intraoral scans, avoiding exposure to radiation.

Furthermore, a study that evaluates rotational movements could be associated with this method, to run a complete 3D evaluation.

## CONCLUSION

Although the proposed method requires three different software to be performed, it proved to be accurate and not dependent on the operator’s calibration. This may be a useful method for tracking translational displacements of 3D structures in a reliable way. Developers could compile the needed tools in a single software in the future, making the workflow more user-friendly and thus stimulating its use by both clinicians and researchers. 
